# Supplementary material for: Identification and functional analysis of non-coding regulatory small RNA FenSr3 in Bacillus amyloliquefaciens LPB-18
Source: PeerJ. 2023 May 15;11:e15236. doi: 10.7717/peerj.15236 (PMC10194069; doi:10.7717/peerj.15236)
Supplement: Supplemental Information 4 [file peerj-11-15236-s004.zip › KO/CK-vs-T1_map/map04115.html]

KEGG PATHWAY: p53 signaling pathway - Reference pathway


|  |  |
| --- | --- |
| **p53 signaling pathway - Reference pathway** |  |

[
Pathway menu
| Organism menu
| Pathway entry
| Show description
| User data mapping
]

p53 activation is induced by a number of stress signals, including DNA damage, oxidative stress and activated oncogenes. The p53 protein is employed as a transcriptional activator of p53-regulated genes. This results in three major outputs; cell cycle arrest, cellular senescence or apoptosis. Other p53-regulated gene functions communicate with adjacent cells, repair the damaged DNA or set up positive and negative feedback loops that enhance or attenuate the functions of the p53 protein and integrate these stress responses with other signal transduction pathways.

|  |  |  |
| --- | --- | --- |
| Reference pathway | 184% 150% 122% 100% 82% 67% 55% | 图片下载 |
